# CGRP-CRLR/RAMP1 signal is important for stress-induced hematopoiesis

**DOI:** 10.1038/s41598-018-36796-0

**Published:** 2019-01-23

**Authors:** Akira Suekane, Yusuke Saito, Shingo Nakahata, Tomonaga Ichikawa, Honami Ogoh, Kazutake Tsujikawa, Kazuhiro Morishita

**Affiliations:** 1 Division of Tumor and Cellular Biochemistry, Department of Medical Sciences, Faculty of Medicine, University of Miyazaki, Miyazaki, Japan; 2 Laboratory of Molecular and Cellular Physiology, Graduate School of Pharmaceutical Sciences, Osaka University, Osaka, Japan

## Abstract

Ecotropic viral integration site-1 (EVI1) has a critical role in normal and malignant hematopoiesis. Since we previously identified high expression of calcitonin receptor like receptor (CRLR) in acute myeloid leukemia (AML) with high EVI1 expression, we here characterized the function of CRLR in hematopoiesis. Since higher expression of CRLR and receptor activity modifying protein 1 (RAMP1) was identified in immature hematopoietic bone marrow (BM) cells, we focused on calcitonin gene-related peptide (CGRP), a specific ligand for the CRLR/RAMP1 complex. To elucidate the role of CGRP in hematopoiesis*, Ramp1*-deficient (*Ramp1*^−/−^) mice were used. The steady-state hematopoiesis was almost maintained in *Ramp1*^−/−^ mice; however, the BM repopulation capacity of *Ramp1*^−/−^ mice was significantly decreased, and the transplanted *Ramp1*^−/−^ BM mononuclear cells had low proliferation capacity with enhanced reactive oxygen species (ROS) production and cell apoptosis. Thus, CGRP is important for maintaining hematopoiesis during temporal exposures with proliferative stress. Moreover, continuous CGRP exposure to mice for two weeks induced a reduction in the number of BM immature hematopoietic cells along with differentiated myeloid cells. Since CGRP is known to be increased under inflammatory conditions to regulate immune responses, hematopoietic exhaustion by continuous CGRP secretion under chronic inflammatory conditions is probably one of the important mechanisms of anti-inflammatory responses.

## Introduction

Hematopoietic stem and progenitor cells (HSPCs) stay in the BM niches that controls survival, self-renewal, or differentiation in the BM^1–8^. When the body is placed under stress conditions, such as a tissue injury, bleeding, inflammation, or DNA damage, stress-induced hematopoiesis occurs, and many cytokines or neural hormones, are secreted to enhance hematopoiesis. The produced granulocytes, monocytes, or lymphocytes may function to resolve these stress events. In the recent literature, HSPCs have been shown to detach from the niche via CXCL12 downregulation and activation of the β3-adrenergic receptor, which results in the enhancement of their proliferation and differentiation, accompanied by an increase in the numbers of neutrophils and monocytes^9,10^. Stress-induced hematopoiesis might be related to one of the survival mechanisms of leukemia stem cells (LSCs)^11,12^.

The transcription factor EVI1 is highly expressed in approximately 10% of AML patients, who mostly present with refractory AML with poor prognosis, and thus, the development of a novel treatment strategy is required^13–18^. Although EVI1 plays an important role in maintaining hematopoietic stem cells (HSCs) and LSCs, the exact molecular functions of EVI1 in stem cells has not been elucidated^19–21^. We recently showed that, *G protein-coupled receptor 56* (*GPR56*), an EVI1-regulated gene, is important for maintaining HSCs and LSCs, along with CD52, integrin α6 and Angiopoietin-1^22-25^.

In this study, we focused on CRLR, which is highly expressed in AML with high EVI1 expression (EVI1^high^ AML)^26^. CRLR forms a complex with not only RAMP1 to serve as a receptor for CGRP, but also RAMP2 or RAMP3 to serve as a receptor for adrenomedullin (ADM)^27^. In the present study, we identified that RAMP1 is specifically expressed in hematopoietic progenitor cells, which led us to speculate that CGRP might be a specific ligand for CRLR in hematopoietic cells. CGRP is a neuropathic peptide that is primarily secreted from sensory nerves after stimulation of transient receptor potential vanilloid receptor-1 (TRPV1), which is distributed in C fibers and upstream, with mechanical stimulation, infection, ischemia, and pain^28–30^. Binding of CGRP to the CRLR/RAMP1 receptor activates adenylate cyclase, which elevates intracellular cAMP and activates protein kinase A (PKA), leading to phosphorylation of multiple targets, such as potassium-sensitive ATP channels, extracellular signal-related kinases (ERKs), and cAMP response element-binding protein (CREB)^28^. Although the ability of CGRP to regulate immune cells, such as dendritic cells and lymphocytes, under inflammation has been reported^29^, the function of CGRP in HSCs and/or LSCs has not been clearly elucidated.

Therefore, here, we investigated how CGRP-mediated activation of the CRLR/RAMP1 signaling pathway affects hematopoiesis using *Ramp1*^−/−^ mice. The CRLR/RAMP1 complex was highly expressed in hematopoietic progenitor cells. The hematopoiesis of *Ramp1*^−/−^ mice under steady-state conditions was not affected, which was revealed by examining their hematopoietic progenitor cells in the BM and differentiated blood cells in the peripheral blood (PB). However, the BM repopulation capacity of *Ramp1*^−/−^ mice was significantly decreased, and the transplanted *Ramp1*^−/−^ bone marrow mononuclear cells (BMMNCs) had low proliferation capacity with enhanced ROS production and cell apoptosis. Therefore, the CGRP-CRLR/RAMP1 signaling pathway might play an important role in hematopoiesis under proliferative stress conditions. Moreover, given that long-term CGRP administration to mice decreased the numbers of most of immature hematopoietic cells and differentiated blood cells, it is suggested that continuous CGRP secretion under inflammation and chronic disease may be involved in the hematopoietic exhaustion.

## Results

### CGRP is a ligand of Crlr/Ramp1 in murine hematopoietic cells and the hematopoiesis of *Ramp1*-deficient mice is maintained under steady-state condition

**Figure 1:**
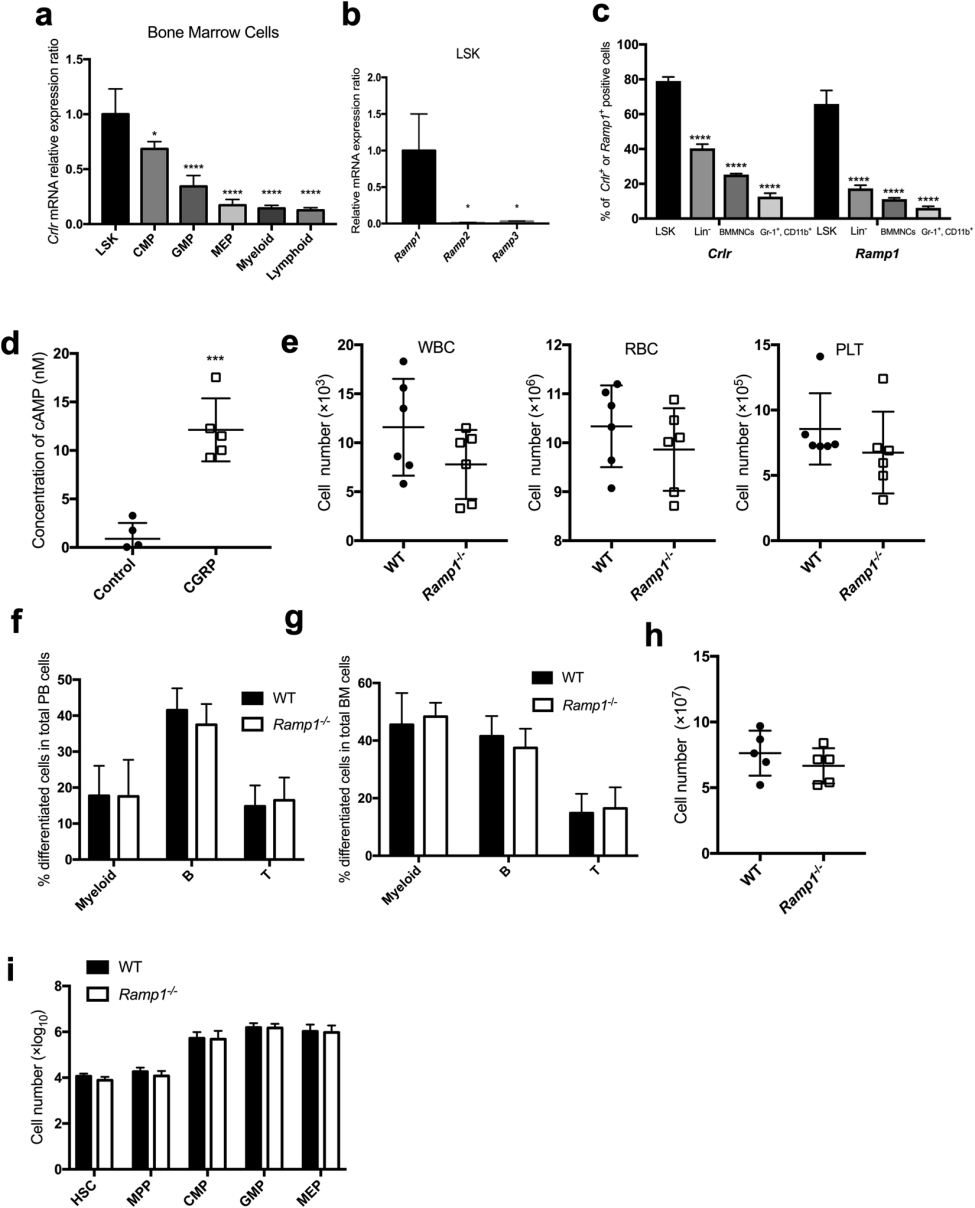
CGRP-Crlr/Ramp1 signal is not an important factor for maintenance of hematopoietic cells under steady-state conditions. (**a**,**b**) Relative expression levels of *Crlr* and *Ramp1* mRNA in purified BM-derived hematopoietic subsets, including LSK, CMP, MEP, GMP, myeloid, and lymphoid cells, as assessed by quantitative real-time RT-PCR. Data are shown as means ± SD for triplicate reactions. *p < 0.05, ****p < 0.0001 compared with LSK or *Ramp1* (Ordinary one-way ANOVA and Dunnett’s multiple comparisons test). (**c**) The percentages of Crlr or Ramp1 positive cells in purified BM-derived hematopoietic subsets, including LSK, lineage negative cells, BMMNCs, and Gr-1^+^CD11b^+^ cells, as assessed by flow cytometry. Data are shown as means ± SD of five mice. ****p < 0.0001 compared with LSK (Ordinary one-way ANOVA and Dunnett’s multiple comparisons test). A representative gating strategy is provided in Supplemental Fig. S1. (**d**) Intracellular cAMP concentrations in LSK cells were measured 10 minutes after treatment with or without 100 nM CGRP. ***p < 0.001 compared with control (Student’s *t* test). (**e**) The WBC, RBC, and PLT counts in the PB of *Ramp1*^−/−^ and WT mice at 8 to 12 weeks of age. (**f**) The percentages of differentiated white blood cells (myeloid cells, B cells, and T cells) in PB from *Ramp1*^−/−^ and WT mice. Data are shown as means ± SD of five mice. (**g**) The percentages of differentiated white blood cells (myeloid cells, B cells, and T cells) in BMMNCs cells from *Ramp1*^−/−^ and WT mice. Data are shown as means ± SD of five mice. (**h**) Absolute numbers of BMMNCs in the BM of *Ramp1*^−/−^ and WT mice. Data are shown as means ± SD of five mice. (**i**) Absolute numbers of immature hematopoietic subpopulations (HSC, MPP, CMP, GMP, and MEP) in the BM of *Ramp1*^−/−^ and WT mice. Data are shown as means ± SD of five mice.

To determine whether Crlr is expressed in normal murine BMMNCs, several BM cell fractions [lineage marker (Lin)^−^ Sca-1^+^ c-kit^+^ (LSK), common myeloid progenitors (CMP) (Lin^−^ Sca-1^−^ c-kit^+^ CD16/32^−^ CD34^+^), granulocyte/monocyte progenitor (GMP) (Lin^−^ Sca-1^−^ c-kit^+^ CD16/32^+^ CD34^+^), megakaryocyte/erythroid progenitor (MEP) (Lin^−^ Sca-1^−^ c-kit^+^ CD16/32^+^ CD34^+^), myeloid (Gr-1^+^CD11b^+^), and lymphoid (B220^+^ for B lineage and CD3e^+^ for T lineage)] were separated from mouse BM by cell sorting using specific antibodies (Methods), and levels of *Crlr* mRNA and protein expression were determined in the BM fractions by real-time PCR (Fig. 1a,b) and flow cytometry (Fig. 1c), respectively. The highest *Crlr* mRNA expression was detected in the LSK fraction, and the expression levels were gradually decreased during progression of differentiation to myeloid and lymphoid lineages (Fig. 1a). Moreover, *Ramp1* mRNA was highly expressed in the LSK fraction, but *Ramp2* and *Ramp3* mRNAs expression was not detected (Fig. 1b), suggesting that the ligand of Crlr is possibly CGRP. The expression patterns of Crlr and Ramp1 proteins were almost same tendency as those of the respective mRNAs (Fig. 1c). To assess whether CGRP is really a ligand for Crlr in hematopoietic progenitor cells, LSK cells were treated with 100 nM CGRP and intracellular cAMP responsiveness was measured (Fig. 1d). The CGRP treatment significantly increased intracellular cAMP concentrations in LSK cells, suggesting that CGRP is a ligand for Crlr and Ramp1 in hematopoietic progenitor cells.

To determine whether the Crlr/Ramp1 signaling pathway affects hematopoietic cell growth and differentiation, *Ramp1*^−/−^ mice were used^31^. *Ramp1*^−/−^ mice were born and grew similarly to wild-type (WT) mice. When mice were 8 to 12 weeks old, we determined the number of blood cells in the PB and BM of the mice. The numbers of white blood cells (WBCs), red blood cells (RBCs), and platelets (Fig. 1e), and percentages of population of myeloid cells (granulocytes and monocytes), and B and T lymphocytes (Fig. 1f) in the PB of *Ramp1*^−/−^ mice were not significantly different from those in WT mice. In addition, percentages of population of myeloid cells, B and T lymphocytes in the BM (Fig. 1g) and the colony-forming ability of BMMNCs (Fig. 1h) were not significantly different between *Ramp1*^−/−^ and WT mice. Moreover, the numbers of BMMNCs and fractionated immature hematopoietic cells with various lineage cell fractions [HSC (Lin^−^ Sca-1^+^ c-kit^+^ CD150^+^ CD48^−^), transiently reconstituting multipotent progenitor (MPP) (Lin^−^ Sca-1^+^ c-kit^+^ CD150^−^ CD48^−^), CMP, GMP, and MEP] were almost the same between *Ramp1*^−/−^ and WT mice (Fig. 1i), suggesting that Crlr/Ramp1 signaling might not an important factor for the regulation of hematopoiesis under steady-state conditions.

### Activation of the Crlr/Ramp1 signaling pathway through CGRP stimulation is important for maintenance of murine hematopoietic stem cells under proliferative stress conditions

**Figure 2:**
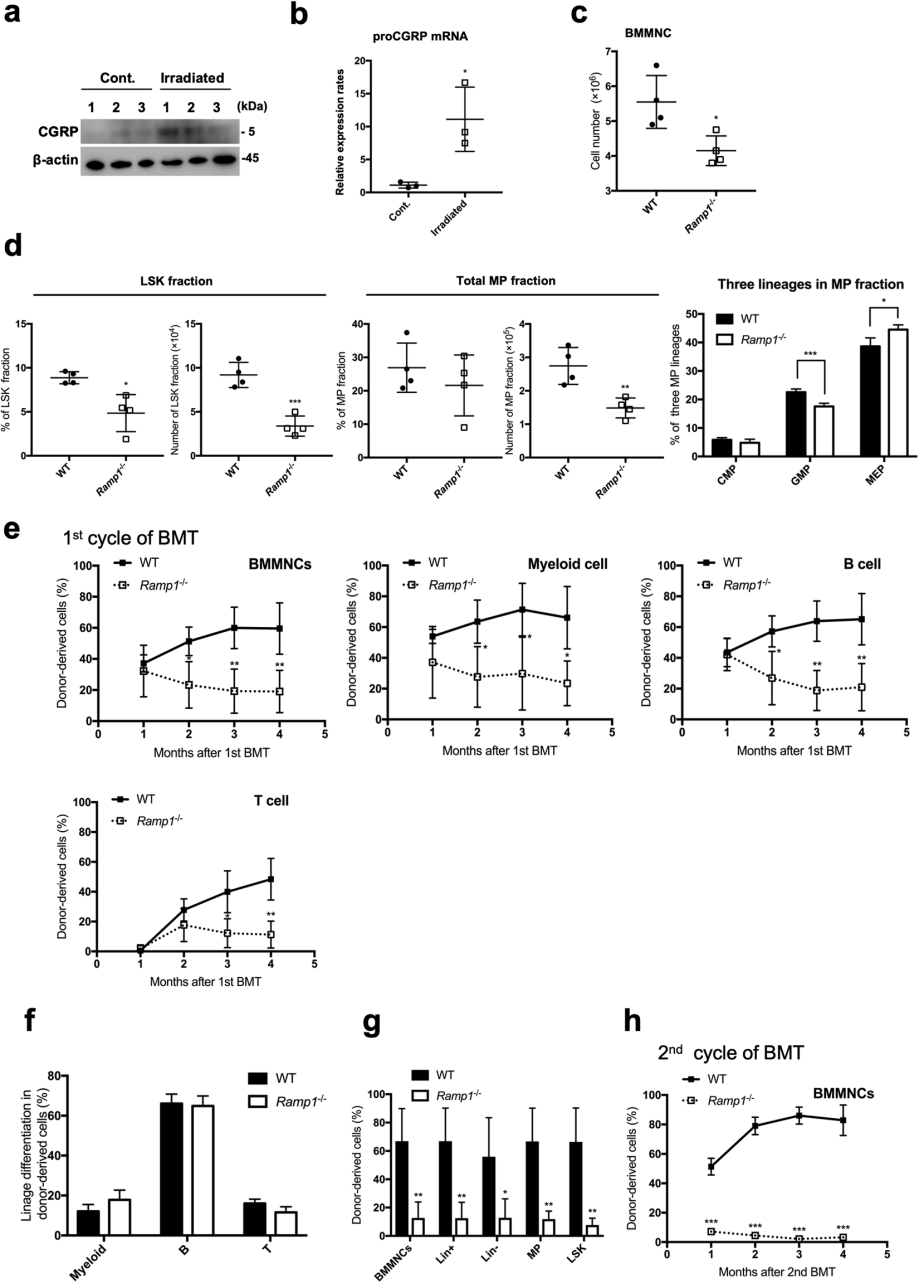
Crlr/Ramp1 signaling is essential for maintenance hematopoietic cells under stress conditions. (**a**) Expression levels of CGRP in BM stromal cells of WT mice with 10 Gy irradiation and without irradiation were determined by immunoblot analysis. BMMNCs from three mice for each group were analyzed. The full-length gels/blots are presented in Supplemental Fig. S4. (**b**) Expression levels of *proCGRP* mRNA were determined in BM stromal cells of WT mice treated with 10 Gy irradiation and without irradiation were determined by real-time PCR. ***p < 0.001 compared with control (Student’s *t* test). (**c**) The numbers of BMMNCs were compared between WT and *Ramp1*^−/−^ mice at seven days after 5-FU injection. Data are shown as means ± SD of four mice. *p < 0.05, compared with WT mice (Student’s *t* test). (**d**) Under the same experiment in (**c**), the cell numbers and percentages of LSK or myeloid progenitors (MP) fractions were compared between WT and *Ramp1*^−/−^ mice at seven days after 5-FU injection. The percentages of CMP, GMP, and MEP cells in the MP fraction were compared between the two groups. Data are shown as means ± SD of four mice. *p < 0.05, **p < 0.01, ***p < 0.001 compared with WT mice (Student’s *t* test). (**e**) Chimerism (percent donor-derived cells, CD45.2) from *Ramp1*^−/−^ and WT mice in BMMNCs, myeloid cells, B lymphocytes, and T lymphocytes from the PB of lethally irradiated WT recipients (CD45.1) at each indicated time point after transplantation (1, 2, 3, and 4 months). Donor/recipient ratio: 50:50. Data are shown as means ± SD of four mice. *p < 0.05, **p < 0.01 compared with WT mice (Student’s *t* test). (**f**) The proportion of donor-derived myeloid, B lymphocytes, and T lymphocytes (*Ramp1*^−/−^ and WT mice) among donor-derived WBCs in the PB of lethally irradiated WT recipients at four months after transplantation. Data are shown as means ± SD of four mice. (**g**) Chimerism (percent donor-derived cells, CD45.2) from *Ramp1*^−/−^ and WT mice in BMMNCs, Lin^+^, Lin^−^, MP, and LSK cells in BMMNCs from lethally irradiated WT recipients (CD45.1). Donor/recipient ratio: 50:50. Data are shown as means ± SD of four mice. *p < 0.05, **p < 0.01 compared with WT mice (Student’s *t* test). (**h**) Chimerism (percent donor-derived cells, CD45.2) from *Ramp1*^−/−^ and WT mice in BMMNCs of lethally irradiated WT recipients (CD45.1) at each indicated time point after second transplantation (1, 2, 3, and 4 months). Donor/recipient ratio: 50:50. Data are shown as means ± SD of three mice. ***p < 0.001 compared with WT mice (Student’s *t* test).

Since CGRP is known to be released from sensory nerves in response to several stress conditions, such as irradiation^32^ or 5-fluorouracil (5-FU) treatment, we here investigated whether Crlr/Ramp1 signaling has an important function in hematopoietic cells under stress conditions using BM transplantation. Initially, to investigate whether radiation therapy induces CGRP secretion in the BM, the BM cells were analyzed for the expression of CGRP protein and *proCGRP* mRNA four days after irradiation with 10 Gy. The levels of CGRP protein and *proCGRP* mRNA were significantly increased after the irradiation treatment (Fig. 2a,b). To evaluate short-term stress hematopoiesis, a single sublethal dose of 150 mg/kg 5-FU was administered to WT (n = 4) and *Ramp1*^−/−^ (n = 4) mice. Since all of the mice survived at 30 days after the 5-FU treatment (Supplemental Fig. S2a), the mice were sacrificed at seven days after 5-FU injection and determined the percentages and absolute numbers of immature BM cells and differentiated myeloid cells. The percentages and total numbers of LSK cells in the BM of *Ramp1*^−/−^ mice were significantly decreased as compared to WT mice (Fig. 2c,d). The numbers of the myeloid progenitor cells (MP; Lin^−^ Sca1^−^ c-kit^+^) and the proportion of GMP were also significantly decreased in the BM of *Ramp1*^−/−^ mice (Fig. 2d) whereas the differentiated myeloid cell population was largely unaffected (Supplemental Fig. S2b). To evaluate hematopoiesis under exposure to temporal proliferative stress, we performed a competitive repopulation assay. Briefly, after 5.0 × 10^5^ of BMMNCs from WT (CD45.2^+^) or *Ramp1*^−/−^ (CD45.2^+^) mice together with the same number of competitor BMMNCs from WT mice (CD45.1^+^) were transplanted into irradiated recipient WT mice (CD45.1^+^), percentages of donor-derived cells in the recipient mice were determined monthly for four months. In the first transplantation, percentages of donor-derived cells in BMMNCs or each PB cell fraction (myeloid, B cells, and T cells) of the recipient *Ramp1*^−/−^ mice were significantly decreased compared with those of WT mice at two months after transplantation (Fig. 2e). Although percentages of population of each differentiated cell lineage in the PB of *Ramp1*^−/−^ mice was not significantly different from those in WT mice four months after transplantation (Fig. 2f), percentages of donor-derived BMMNCs, Lin^+^, Lin^−^, Myeloid progenitor (MP), and LSK cells in the *Ramp1*^−/−^ BMMNCs-transplanted mice were significantly decreased compared with those in WT mice (Fig. 2g). Furthermore, the second round of transplantation using the BMMNCs collected from recipient mice was performed under the same conditions shown in Fig. 2e. The percentage of donor-derived BMMNCs from *Ramp1*^−/−^ mice was significantly decreased compared with that of WT mice (Fig. 2h). Therefore, although the differentiation ability of BM cells in the *Ramp1*-deficient mice was preserved, the repopulation ability of the progenitor cells was significantly decreased in the *Ramp1*^−/−^ mice. Thus, the results suggest that the Crlr/Ramp1 signaling pathway in progenitor cells stimulated with CGRP might be important for maintenance of hematopoietic stem cells during temporal exposures with proliferative stress.

### Enhanced cell proliferation with a reduction in ROS production and apoptosis of HSPCs under proliferative stress conditions by CGRP stimulation

**Figure 3:**
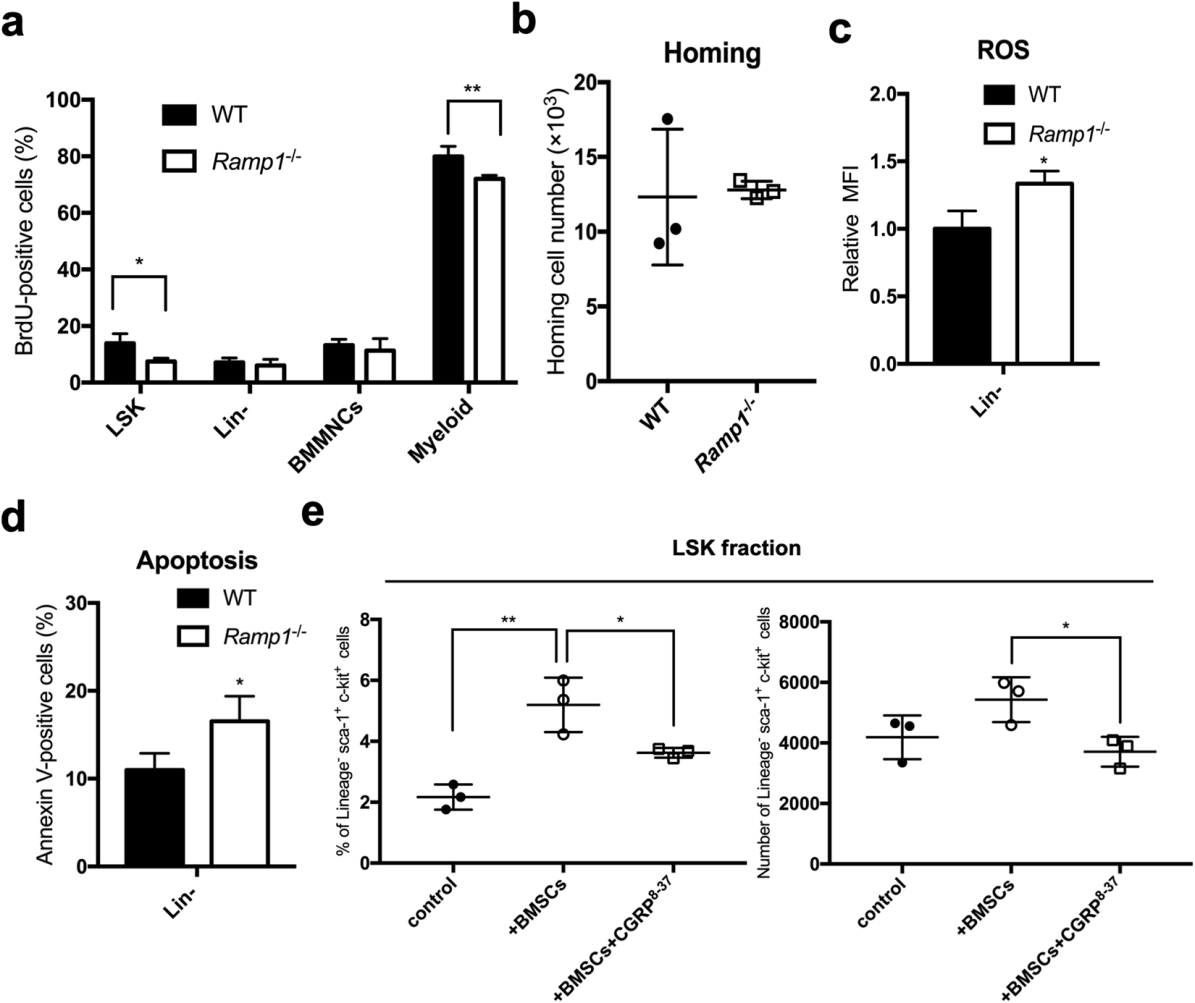
CGRP enhances cell proliferation with suppression of ROS production and cell apoptosis in HSPCs under stress conditions. (**a**) Percentages of BrdU-positive cells in each BM cell fraction (LSK, Lin^−^, BMMNCs, and myeloid cells) of the recipient mice (CD45.1) following transplantation of BM cells (CD45.2) from *Ramp1*^−/−^ and WT mice were determined by flow cytometry. Data are shown as means ± SD of three mice. *p < 0.05, **p < 0.01 compared with WT mice (Student’s *t* test) (**b**) The homing ability of the transplanted donor BMMNCs (*Ramp1*^−/−^ and WT) into the BM of recipient mice was determined by flow cytometry. Total numbers of donor-derived CD45.2^+^ BMMNCs in each recipient mouse are shown. (**c**) The ROS production in Lin^−^ cells from BMMNCs of the recipient mice transplanted with donor BM cells from *Ramp1*^−/−^ or WT mice was determined by flow cytometry using redox-sensitive MitoTracker Orange CMH2TMROS fluorescence staining 16 hours after transplantation. Data are shown as means ± SD of three mice. *p < 0.05 compared with WT mice (Student’s *t* test). (**d**) The percentage of apoptotic cells in Lin^−^ cells from BMMNCs of the recipient mice transplanted with donor BM cells from *Ramp1*^−/−^ or WT mice were determined by flow cytometry using annexin V staining after transplantation. Data are shown as means ± SD of three mice. *p < 0.05 compared with WT mice (Student’s *t* test). (**e**) The percentages and absolute numbers of LSK cells in the Lineage negative population from BMMNCs cultured alone or co-cultured with BM stromal cells in the presence or absence of CGRP^8–36^, as determined by flow cytometery. Data are shown as means ± SD of three mice. *p < 0.05, **p < 0.01 compared with WT mice (Ordinary one-way ANOVA and Turkey’s multiple comparisons test).

To examine how the Crlr/Ramp1 signaling pathway functions in hematopoietic cells under the proliferative stress conditions, we determined the cell proliferation, ROS production, and cell apoptosis in BM transplantation experiments. 24 hours after transplantation of 1.0 × 10^7^ of donor BMMNCs from WT (CD45.2^+^) or *Ramp1*-deficient mice (CD45.2^+^) into irradiated recipient mice (CD45.1^+^), we determined the percentage of population of 5-bromo-2′-deoxyuridine (BrdU)-positive cells in the BM cell fraction. Percentages of population of BrdU-positive cells in LSK and Gr-1^+^CD11b^+^ myeloid cell fraction were significantly decreased by a *Ramp1* deficiency (Fig. 3a), although the homing abilities of the transplanted BMMNCs were not significantly different between *Ramp1*^−/−^ and WT mice (Fig. 3b**)**. Moreover, the rate of ROS production and the percentage of apoptotic cells were significantly increased in Lin^−^ cells from *Ramp1*^−/−^ mice as compared to those in WT mice (Fig. 3c and d). To evaluate whether intrinsic CGRP secretion from mesenchymal cells affect the growth and differentiation of BM cells under stress conditions, the BMMNCs isolated from WT mice were cultured alone or co-cultured with BM stromal cell fraction in the presence or absence of CGRP^8–36^, a peptide antagonist for CGRP1 receptor. After co-culturing with BM stromal cells, the numbers of LSK cells were significantly increased, while LSK numbers were unchanged in the presence of CGRP^8–36^ (Fig. 3e), suggesting that the CGRP signaling from BM stromal cells is essential for the increase of LSK pool in the BM. Additionally, the numbers of differentiated myeloid cells were not significantly different between the three groups under these conditions (Supplemental Fig. S3). Therefore, these results suggest that the secretion of CGRP from mesenchymal cells might promote the hematopoiesis through the enhancement of cell proliferation with suppression of ROS production and apoptosis in BM cells under proliferative stress conditions.

### Sustained CGRP treatment leads to the reduction in the number of hematopoietic cells *in vivo*

**Figure 4:**
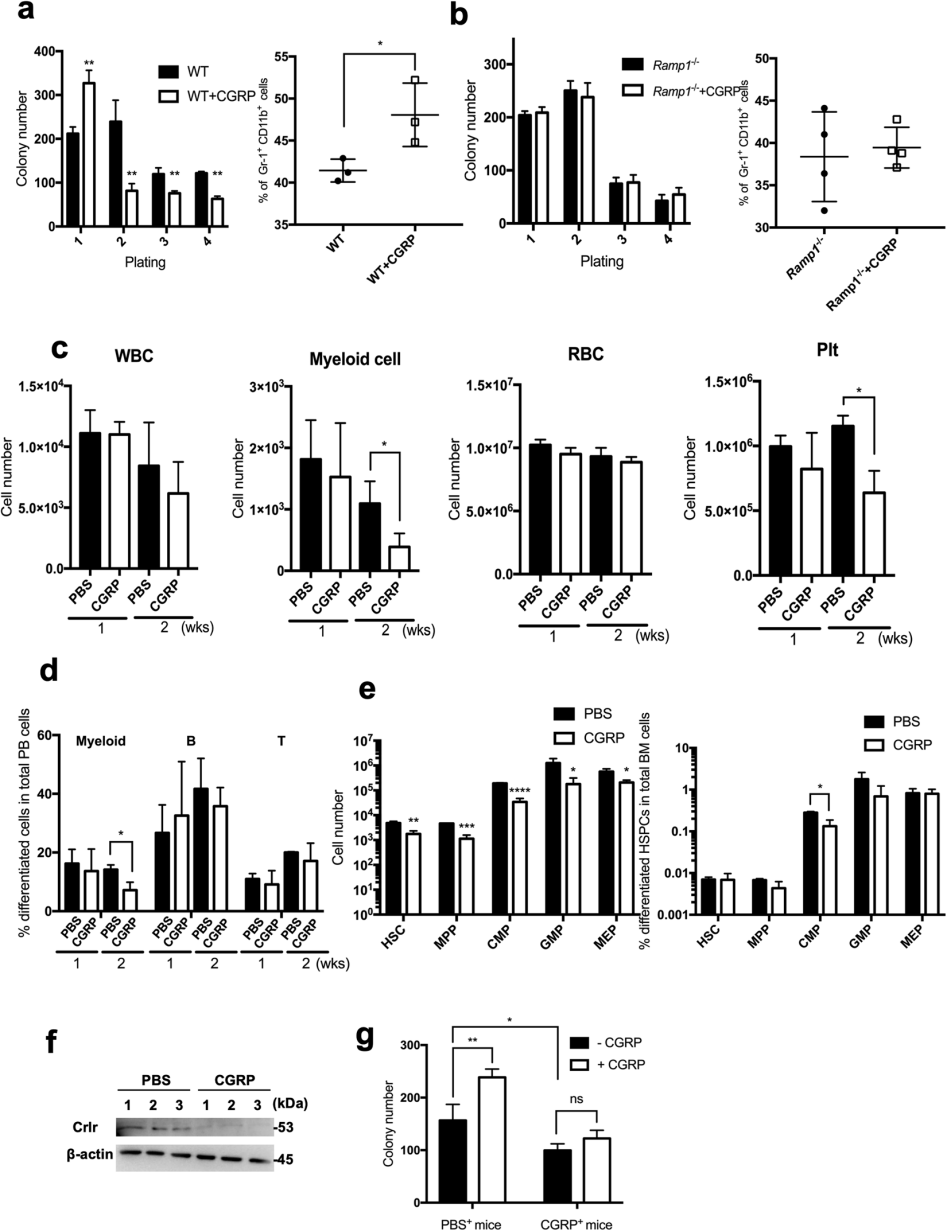
Long-term CGRP treatment affects hematopoiesis. (**a**) The colony-forming abilities of BMMNCs (left) and the percentage of Gr-1^+^CD11b^+^ cells in BMMNCs (right) from WT mice were determined by serial plating in methylcellulose culture with or without CGRP (100 nM). Data are shown as means ± SD of three mice. *p < 0.05, **p < 0.01 compared with WT mice (Student’s *t* test). (**b**) The colony-forming abilities of BMMNCs (left) and the percentage of Gr-1^+^CD11b^+^ cells in BMMNCs (right) from *Ramp1*^−/−^ mice were determined by serial plating in methylcellulose culture with or without CGRP (100 nM). Data are shown as means ± SD of three mice. (**c**) The WBC, myeloid cells, RBC, and Plt counts in the PB of WT mice treated with CGRP or PBS for one and two weeks were determined. Data are shown as means ± SD of three mice. *p < 0.05 compared with PBS-treated mice (Student’s *t* test). (**d**) The percentages of differentiated white blood cells populations (myeloid cells, B cells, and T cells) in PB cells from WT mice treated with CGRP or PBS for two weeks were determined. Data are shown as means ± SD of three mice. *p < 0.05 compared with PBS-treated mice (Student’s *t* test). (**e**) The cell numbers (left) and percentages (right) of hematopoietic progenitor subpopulations (HSC, MPP, CMP, GMP, and MEP) in BMMNCs of WT mice treated with CGRP or PBS for two weeks were determined. Data are shown as means ± SD of three mice. *p < 0.05, **p < 0.01, ***p < 0.001, ****p < 0.0001 compared with PBS-treated mice (Student’s *t* test). (**f**) Expression levels of Crlr in BMMNCs of WT mice treated with CGRP or PBS for two weeks was determined by immunoblot analysis. BMMNCs from three mice for each group were analyzed. The full-length gels/blots are presented in Supplemental Fig. S4. (**g**) The colony forming abilities of BMMNCs of PBS-treated (PBS^+^) or CGRP- treated (CGRP^+^) mice for two weeks were determined in the *in vitro* cell culture with (CGRP) or without CGRP (Control). BMMNCs from three mice for each group were analyzed. ns; not significant, *p < 0.05, **p < 0.01 compared with PBS-treated mice (Ordinary one-way ANOVA and Dunnett’s multiple comparisons test).

To evaluate whether continuous CGRP treatment affects hematopoietic cells*, in vitro* replating assays were performed in culture medium with or without CGRP. The colony-forming ability and the percentage of Gr-1^+^CD11b^+^ myeloid cell population in BMMNCs from WT mice were significantly increased in culture medium with CGRP as compared to those of the medium without CGRP in the first replating (Fig. 4a). However, the colony-forming ability was significantly reduced in the presence of CGRP after the second replating (Fig. 4a, left). In addition, the colony-forming ability and the percentage of myeloid cell population in BMMNCs from *Ramp1*^−/−^ mice were not significantly different between CGRP-treated and untreated groups (Fig. 4b). These results suggest that CGRP treatment enhances the colony-forming ability of BMMNCs with myeloid cell differentiation, and that continuous stimulation with CGRP may reduce the hematopoietic proliferation and differentiation of BM cells.

To examine whether continuous CGRP exposure affects hematopoietic cells *in vivo*, high-dose CGRP (0.2 mg/two weeks) or phosphate-buffered saline (PBS) as a control were subcutaneously and continuously administered into WT mice for two weeks using an osmotic infusion pump, and the numbers of blood cells and the percentages of population of each PB cell fraction (myeloid, B cells, and T cells) were determined at one and two weeks after CGRP treatment. One week after the CGRP treatment, the numbers of WBCs, myeloid cells, RBCs, and PLTs were not significantly different between the control and CGRP-treated groups; however, the numbers of myeloid cells and PLTs were significantly reduced two weeks after the CGRP treatment (Fig. 4c). In addition, within the WBC population, the percentages of myeloid cell population were significantly decreased (Fig. 4d), suggesting that the population of differentiated myeloid cells might be depleted during two weeks of continuous CGRP treatment. Moreover, among BMMNCs, the numbers of all immature hematopoietic cell fraction including HSC and CMP, were significantly decreased in CGRP-treated mice as compared to those of the controls (Fig. 4e, left), while the percentage of CMP was significantly decreased in CGRP-treated mice than in controls (Fig. 4e, right). To study the reduction mechanism of blood cells after two weeks of continuous CGRP treatment, the expression of Crlr in BMMNCs cells was determined by immunoblot analysis. As shown in Fig. 4f, the Crlr expression was decreased in BMMNCs after two-weeks treatment with CGRP. Moreover, after separation of BMMNCs from CGRP-treated mice, we determined the colony-forming abilities of BMMNCs in the presence or absence of CGRP in *in vitro* culture. In the absence of CGRP, the colony-forming abilities of BMMNCs from CGRP-treated mice were significantly decreased compared to those of WT-mice (Fig. 4g). While the CGRP treatment significantly increased the colony-forming abilities of BMMNCs from the PBS-treated mice, it had no obvious effect on the colony-forming abilities in BMMNCs from the CGRP-treated mice (Fig. 4g). These results suggest that continuous exposure of CGRP induces reduction of myeloid/erythroid/megakaryocyte immature hematopoietic progenitor cells with downregulation of the Crlr expression in BM cells, leading to the depletion of immature and differentiated blood cells, particularly myeloid cells.

## Discussion

In this study, we identified CGRP as the specific ligand for CRLR/RAMP1 in HSPCs. We found that the CGRP-CRLR signaling is not an important factor for the regulation of murine hematopoiesis under steady-state conditions, and that the BM repopulation ability of BMMNCs from *Ramp1*-deficient mice is significantly inhibited following BM transplantation in mice. Moreover, the transplanted BMMNCs from *Ramp1*-deficient mice displayed a decreased cell proliferation with enhanced ROS accumulation and apoptosis, while CGRP treatment of BMMNCs from wild-type mice but not *Ramp1*-deficient mice increased the colony forming ability along with production of differentiated progenitor cells. Surprisingly, however, continuous treatment with CGRP induced a reduction of hematopoietic proliferation and differentiation *in vivo* in mice as well as in *in vitro* experiments. Therefore, we propose that one of the important functions of CGRP in HSPCs is possibly to enhance the hematopoiesis under temporal exposures to proliferative stress; however, under pathological conditions of persistent proliferative stress via continuous CGRP secretion, HSPCs lose Crlr expression, which may promote the depletion of hematopoietic progenitors and differentiated cells.

CGRP is one of the main neurotransmitters involved in immune function and is released in response to activation of TRPV1 through sensory neurons and C fibers and is a key responder to tissue damage that is perceived as “pain”. The sensory C fiber neurons innervate every organ in the body and particularly TRPV1 and CGRP have been identified in the subset of neuron fibers within the trabecular bone in the BM^33^. When TRPV1 is activated by external factors, such as heat, pH, ischemia, bone fracture, irradiation, or toxins, CGRP is released from the sensory neurons, resulting in the expansion of the numbers of circulating granulocytes, macrophages, mast cells, and other immune cells at various locations to defend the body and heal the damage. The CGRP signaling has been implicated in the cAMP, PKC, ERK, and p38 signal transduction pathways^28^. In particular, cAMP-dependent activation of CREB transcription factor has been shown to play a central role in granulopoiesis directly from CD34^+^ BM cells^34^. In this study, we demonstrated for the first time that cells of the BM stromal fraction secretes CGRP to stimulate proliferation and differentiation of BM cells, and that the CGRP-CRLR/Ramp1 signaling plays a critical role in stress-induced hematopoiesis.

CGRP has an important function that connects nervous and immune systems, and directly or indirectly targets peripheral immune cells such as dendritic cells, mast cells, T cells and macrophages in peripheral tissues^29^. In acute stages of inflammation, CGRP that is present in peripheral tissues is known to enhance inflammatory responses by dilation of blood vessels, extravasation of inflammatory cells, activation of secretion of inflammatory cytokines^35^, and, in the later stage of inflammation, the proinflammatory activities of macrophages and lymphocytes can be inhibited by the action of CGRP, leading to the suppression of inflammatory responses^36^. Consistent with this, CGRP stimulates hematopoiesis in the BM after initiation of inflammation, which may compensate proliferating hematopoietic cells including monocytes, which are recruited to the local inflammatory tissues. On the other hand, in chronic inflammation, expression of the CGRP receptor CRLR may be reduced and the loss of CGRP function may inhibit hematopoiesis with reduction of myeloid cells, which could terminate the inflammatory responses by reduction of tissue-invaded microphages.

In summary, the CGRP-CRLR/RAMP1 signaling acts to promote hematopoiesis under proliferative stress conditions, but the sustained CGRP secretion conversely reduces undifferentiated BM cells together with differentiated myeloid cells. Therefore, these results suggest that CGRP may play an important role in biological defenses including infection and inflammation by integrating nervous system, hematopoiesis, and immunity.

## Methods

### Mice

All animal experiments were performed in accordance with protocols approved by the Animal Experiment Review Board of the University of Miyazaki. *Ramp1*-deficient mice were described elsewhere^31^. C57BL/6 CD45.1 congenic mice (B6-CD45.1) were purchased from Sankyo-Lab Service (Tokyo, Japan). For analysis of blood counts, PB from the tail vein was collected in EDTA-coated microtubes and analyzed with a hematology analyzer (CellTac, NIHON KOHDEN, Tokyo, Japan).

### 5-FU treatment in mice

A single dose of 5-fluorouracil (5-FU) at 150 mg/kg was administered by intraperitoneal injection to 8–12 weeks old WT (n = 3) and *Ramp1-*deficient (n = 3) mice and the mice were followed up until 30 days. In the second experiment, the treated mice (n = 4 in each group) were sacrificed at seven days after injection and populations of LSK cells (Lin^−^ Sca-1^+^ c-kit^+^), myeloid progenitor cells (MP; Lin^−^ Sca-1^−^ c-kit^+^) including CMP, GMP, and MEP, and BMMNCs were analyzed by flow cytometry.

### CGRP administration in mice

A total of 0.2 mg of CGRP in physiological saline was infused into the subcutaneous tissues of the backs of mice using osmotic pumps (Alzat, Cupertino, CA, USA) at 0.25 l/h rate, and the administration was continued for 14 days.

### Long-term competitive repopulation assay

C57BL/6-CD45.1 mice and *Ramp1*^−/−^ or WT/*Ramp1*^+/+^-CD45.2 mice were used for the long-term competitive repopulation assay, according to the procedure previously described^37^. BM cells were isolated from tibia and femoral bones of 8–10 weeks old *Ramp1*^−/−^ or WT/*Ramp1*^+/+^-CD45.2 mice. Eight to 10 weeks old C57BL/6-CD45.1 mice were irradiated individually with a lethal dose of 10 Gy two times (3 hours interval) and prepared as recipient mice. 5 × 10^5^ BMMNCs obtained from *Ramp1*^−/−^ or WT/*Ramp1*^+/+^-CD45.2 mice were mixed with 5 × 10^5^ BMMNCs from C57BL/6-CD45.1 mice and were transplanted into lethally irradiated C57BL/6-CD45.1 mice intravenously. Every four weeks after BM transplantation, CD45.2 donor cell repopulation in the PB was monitored for at least 16 weeks by flow cytometry using the CD45.1, CD45.2 and lineage antibodies. BMMNCs from primary recipient were transplanted in secondary recipient CD45.1 mice and the PB was monitored in a manner same to that in primary recipient.

### Analysis of *in vivo* cell proliferation

BM cells were isolated from tibia and femoral bones of 8–10 weeks old *Ramp1*^−/−^ or WT/*Ramp1*^+/+^-CD45.2 mice. Eight to 10 weeks old C57BL/6-CD45.1 mice were irradiated individually with a lethal dose of 10 Gy two times (3 hours interval) and prepared as recipient mice. 1 × 10^7^ BMMNCs obtained from *Ramp1*^−/−^ or WT/*Ramp1*^+/+^-CD45.2 mice were transplanted into lethally irradiated C57BL/6-CD45.1 mice intravenously. After transplantation, mice were given three intraperitoneal injections of 1 mg of BrdU (8 hour interval). 24 hours after transplantation, BrdU incorporation *in vivo* was measured via flow cytometry using an APC BrdU Flow Kit (BD Biosciences, San Jose, CA, USA) according to the manufacturer’s instructions.

### Co-culture of BMNNCs with BM stromal cells

BM stromal cells were isolated from tibia and femoral bones of 8–10 weeks old mice, according to the procedure previously described^38^. Briefly, intact bones were crushed in PBS with mortar and pestle and washed three times with HSBB + buffer [2% fetal bovine serum (FBS), 10 mM HEPES, penicillin-streptomycin] to release marrow containing BMMNCs. Marrow was reserved at room temperature, while bone fragments were cut into small fragments, and incubated with collagenase at 37 °C for 60 min to obtain the bone-associated cells. The digested mixture containing adherent mesenchymal stromal cells was then filtered through a 70 μm strainer (BD Bioscience, San Jose, CA, USA), centrifuged at 280 × g at 4 °C, and resuspended in HSBB+. To isolate BMMNCs, the marrow suspensions were treated with ACK lysing buffer (0.155 M ammonium chloride, 0.1 M disodium EDTA and 0.01 M potassium bicarbonate) to lyse erythrocytes and washed with 2 × PBS containing 4% FBS, and the cell suspensions were filtered through a 70 μm strainer (BD Bioscience) and resuspended in HSBB+. 1.0 × 10^6^ of BMMNCs were cultured alone or with the BM stromal cells (1.0 × 10^6^) in α-MEM medium supplemented with 10 ng/ml each of stem cell factor (SCF), thrombopoietin (TPO), and Flt3 with or without 100 nM CGRP^8–36^, a peptide antagonist for Crlr/Ramp1 receptor. After culturing for two weeks, the percentage of LSK and Gr-1^+^CD11b^+^ fraction by flow cytometry.

### Analysis of CGRP expression after irradiation in BM stromal cells

Four days after 10 Gy total body irradiation to 8–10 weeks old mice, the BM stromal cells were isolated from tibia and femoral bones of the irradiated mice^38^. The CGRP protein and *proCGRP* mRNA in the BM stromal cells from the mice with (Irradiated) or without irradiation (Cont.) were determined by immune blot and real-time PCR, respectively.

### Colony assay

BM cells were seeded at a density of 1 × 10^4^ cells/well in a 12-well plate in semisolid methylcellulose-based medium (MethoCult M3434, Stem Cell Technologies, Vancouver, Canada) in duplicate according to the manufacturer’s instructions. After 7 days of culture, the total number of colonies was determined under an inverted microscope (Olympus, Tokyo, Japan). The cultured cells were then washed with PBS, and 1 × 10^4^ cells were replated in MethoCult M3434 medium; colonies were counted 7 days after replating. This procedure was repeated four times. The differentiation status was assessed by flow cytometry analysis.

### Flow cytometry

BM cells were collected from femurs and tibiae. The other mononuclear cells were collected from the PB and spleen. Flow cytometry and cell sorting were performed using a JSAN cell sorter (Bay bioscience, Kobe, Japan). The ROS levels were measured using CellROX Deep Red Reagent (Life Technologies, Carlsbad, CA, USA) according to the manufacturer’s protocol. For apoptosis analysis, cells were stained using an Annexin V Apoptosis Detection Kit I (BD Biosciences-Pharmingen, Franklin Lakes, NJ, USA) following the manufacturer’s instructions. To determine the population of differentiated white blood cells, white blood cells were separated into myeloid cells (CD11b^+^Gr-1^+^), B lymphocytes (B220^+^), and T lymphocytes (CD4^+^) using flow cytometry.

### Antibodies

The monoclonal antibodies used in this study targeted the following proteins: CRLR (H-42; Santa Cruz, Dallas, TX, USA), RAMP-1 (EPR10867; abcam, Cambridge, UK), Gr-1 (RB6-8C5; BioLegend, San Diego, CA, USA), Mac-1 (M1/70; BioLegend), B220 (RA3-6B2; BioLegend), CD3e (145-2C11; Biolegend), CD2 (RM2-5; TONBO, Japan), CD8a (53–6.72; TONBO), TER-119 (TER-119; TONBO), CD127 (A7R34; TONBO), c-Kit (2B8; BioLegend), Sca-1 (D7; BioLegend), CD34 (RAM34; eBioscience, Santa Clara, CA, USA), CD16/32 (93; eBioscience), CD48 (HM48-1; BioLegend), CD150 (TC15–12F12.2; BioLegend), CD45.2 (104; eBioscience) and CD45.1 (A20; eBioscience). A mixture of mAbs against CD2, CD3e, CD8a, B220, TER-119, CD127, Mac-1 and Gr-1 served as lineage markers (Lineage). Mouse anti-BrdU-Alexa Fluor 647 antibody (3D4; BD Biosciences) was used to detect intracellular BrdU.

### Western blotting

For protein extraction from tissues, excised organs were homogenized in NP-40 lysis buffer (50 mM Tris-HCl, pH8.0, 150 mM NaCl, 5 mM EDTA, and 1% NP-40) supplemented with a proteinase inhibitor cocktail (Sigma-Aldrich, St. Louis, MO, USA) and phosphatase inhibitor tablet (Roche, Penzberg, Germany). The lysate was centrifuged for 10 minutes at 15000 × g (maximum) and 4 °C, and the supernatant was then collected. The protein concentration was determined with a BCA protein assay (Thermo Scientific, Waltham, MA, USA), with bovine serum albumin (BSA) as the standard. Equal amounts of protein samples were loaded into SDS-polyacrylamide gels for electrophoresis, and then, the separated proteins were transferred to a polyvinylidene difluoride membrane (Millipore, Billerrica, MA, USA). The membranes were blocked in PBS–Tween (0.1%) (PBST) with 1% BSA or 5% nonfat dried milk and were then probed with the primary antibodies diluted PBST-BSA, 5% nonfat dried milk or Can Get Signal Buffer (TOYOBO, Osaka, Japan). The bands were detected using a Lumi-light Plus kit (Roche, Penzberg, Germany) and LAS-3000. All primary antibodies were used at a dilution of 1:1000.

### Quantitative real-time RT-PCR

Total RNA was extracted from cells using TRIzol reagent (Thermo Fisher Scientific, Waltham, MA), and 1 µg of total RNA was reverse transcribed to obtain first-strand cDNA using an RNA-PCR kit (Takara-Bio Inc., Shiga, Japan). Quantitative real-time RT-PCR was performed with GeneAce SYBR qPCR Mix a (Nippon Gene, Toyama, Japan) using a StepOne Real-time PCR System (Applied Biosystems, Waltham, MA, USA). The amplification data were analyzed with StepOne software (Applied Biosystems). PCR was performed in triplicate and the expression level was normalized to the expression of β-actin. All the primers used are listed in Supplemental Table S1.

### Statistics

Statistics were calculated using GraphPad Prism 7 software (Graphpad, San Diego, CA, USA). Unpaired 2-tailed Student’s *t* tests were used when 2 groups were compared. Ordinary one-way ANOVA and Dunnett’s or Turkey’s multiple comparisons test were used for multiple comparisons.

## Supplementary information

Supplementary information
