# Supplementary material for: CGRP-CRLR/RAMP1 signal is important for stress-induced hematopoiesis
Source: Sci Rep. 2019 Jan 23;9:429. doi: 10.1038/s41598-018-36796-0 (PMC6344543; doi:10.1038/s41598-018-36796-0)
Supplement: Supplementary file 1 — Supplementary information [file 41598_2018_36796_MOESM1_ESM.docx]

Supplementary Information for

**CGRP-CRLR/RAMP1 signal is important for stress-induced hematopoiesis**

Akira Suekane^1^, Yusuke Saito^1^, Shingo Nakahata^1^, Tomonaga Ichikawa^1^, Honami Ogoh^1^, Kazutake Tsujikawa^2^, Kazuhiro Morishita^1^

^1^Division of Tumor and Cellular Biochemistry, Department of Medical Sciences, Faculty of Medicine, University of Miyazaki, Miyazaki, Japan

^2^Laboratory of Molecular and Cellular Physiology, Graduate School of Pharmaceutical Sciences, Osaka University, Osaka, Japan

To whom correspondence should be addressed. E-mail: kmorishi@med.miyazaki-u.ac.jp

**Supplemental Table S1**

**Supplemental Figure S1-4**

**Supplemental Reference**

**Supplemental Tables**

**Supplemental Table S1. List of the primers for qRT-PCR**

| Species | Gene | Forward primer (5'-3') | Reverse primer (5'-3') |
| --- | --- | --- | --- |
| mouse | Crlr | CAAGATCATGACGGCTCAATA | CGTCATTCCAGCATAGCCAT |
| mouse | Ramp1 | CCTCTGCTTACCTCTGAGATTG | ATCTGTGCAGTCTTCCTTGGAGT |
| mouse | Ramp2 | GTCCATGCAACTCTTGTACTCATACC | GTCCATGCAACTCTTGTACTCATACC |
| mouse | Ramp3 | GGTCATTAGGAGCCACGTGT | GGGCTAAACAAGCCACAGCT |
| mouse | proCGRP^1^ | CCCCAGAATGAAGGTTACACA | TGTCAAAGGGAGAAGGGTTTT |
| mouse | β-actin | TTCTACAATGAGCTGCGTGTG | GGGGTGTTGAAGGTCTCAAA |

**Supplemental Figure S1**


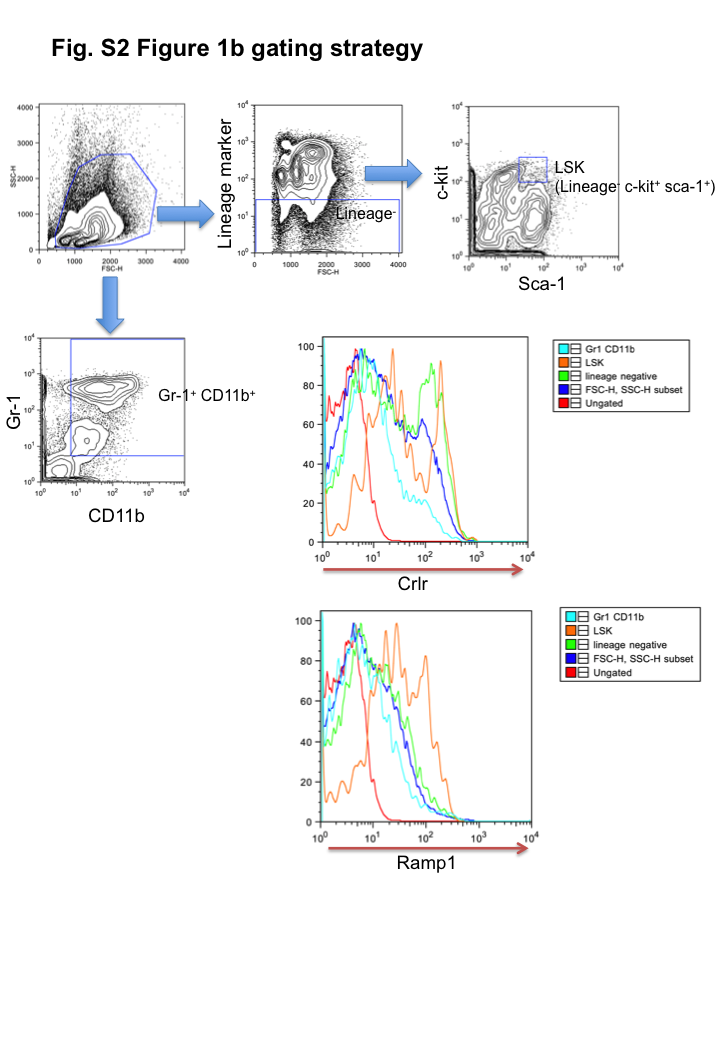


**Supplemental Figure S1. Gating strategy for LSK cells and myeloid cells.** Representative FACS strategy used to isolate LSK and Gr-1^+^CD11b^+^ myeloid cell fractions from BMMNCs. Histogram plots showing Crlr or Ramp1 expression from each cell fraction.

**Supplemental Figure S2a**


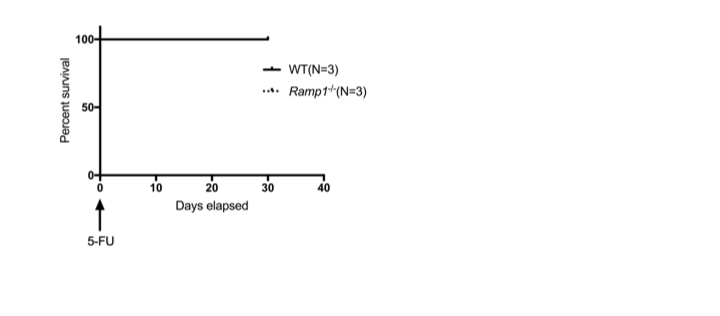


**Supplementary Figure S2b**


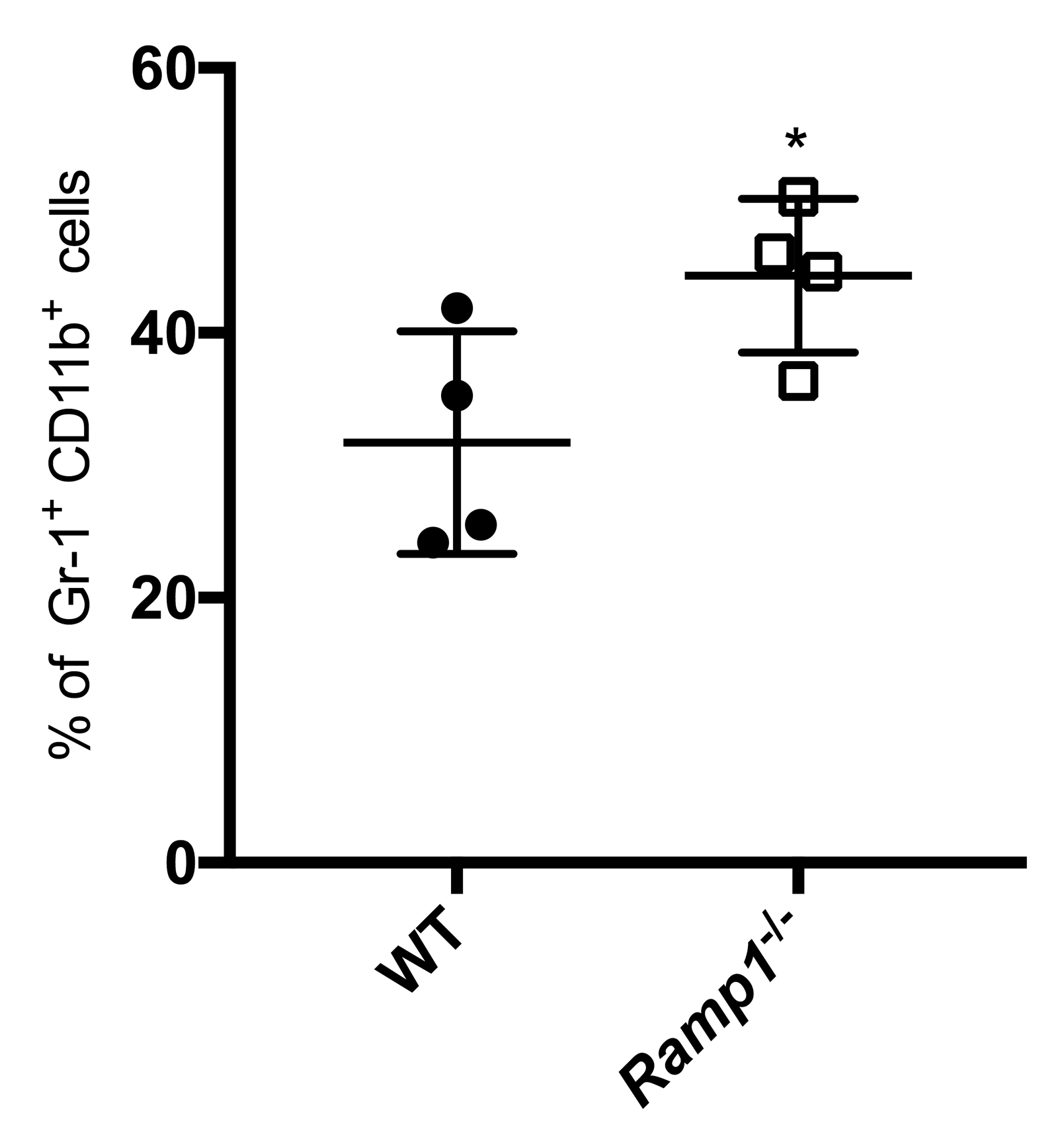

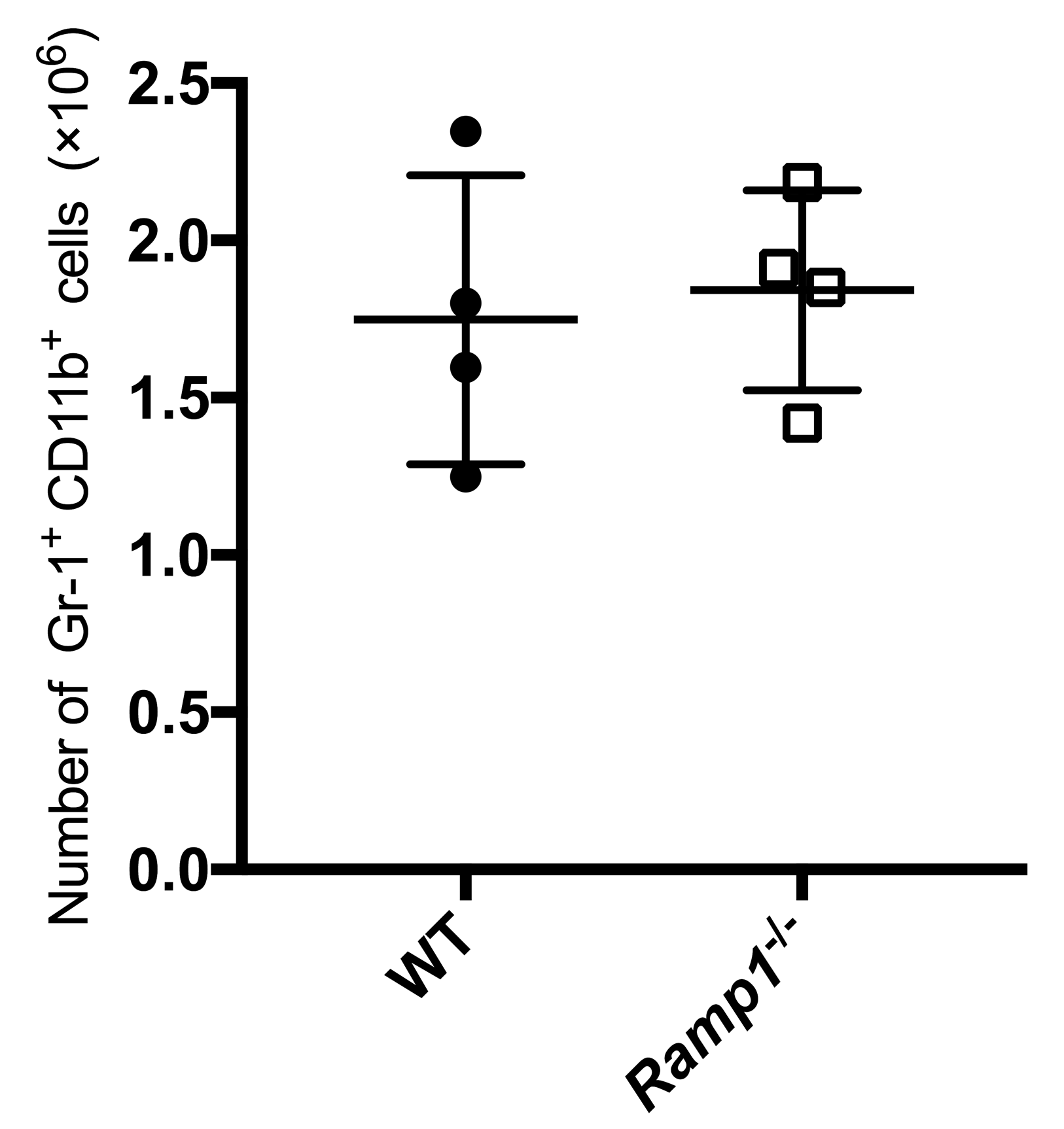


**Supplemental Figure S2.** **Crlr/Ramp1 signaling plays an important role in short-term stress hematopoiesis.**

(**a**) Kaplan Meier survival analysis curves of WT and *Ramp1^-/-^* mice treated with 5-FU. Survival of mice was monitored after single administration of sublethal dose (150 mg/kg) of 5-FU. Three mice per each group were analyzed. Note that all mice survived 30 days in both groups.

(**b**) Flow cytometry analysis of differentiated myeloid cells in the BMMNCs at seven days after 5-FU treatment. The cell numbers and percentages of Gr-1^+^CD11b^+^ cell fraction in the BMNNCs were compared between WT and *Ramp1*-deficient *(Ramp1^-/-^*) mice. Data are shown as means ± SD of four mice. **p* < 0.05, compared with WT mice (Student's t test).

**Supplemental Figure S3**


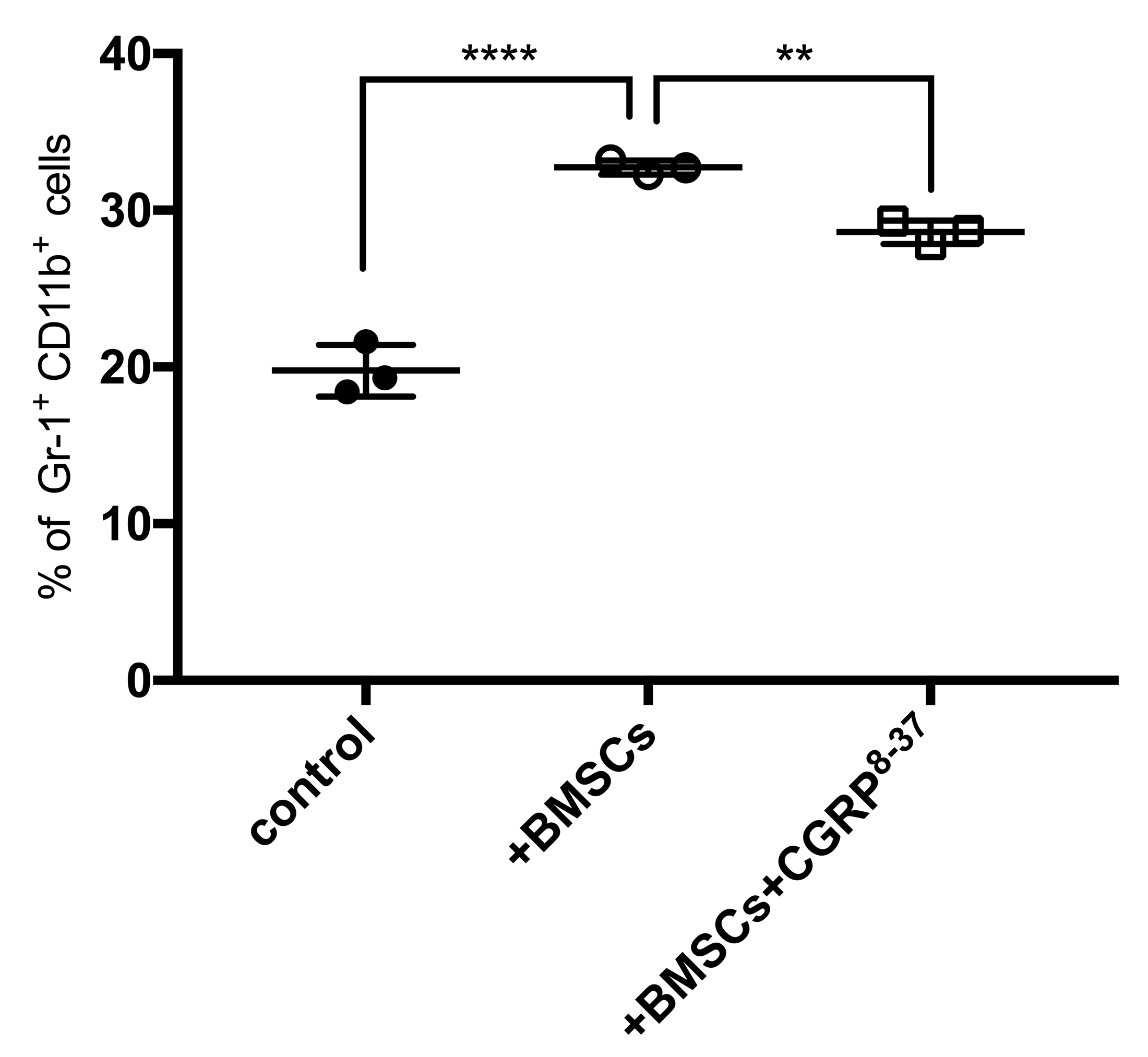

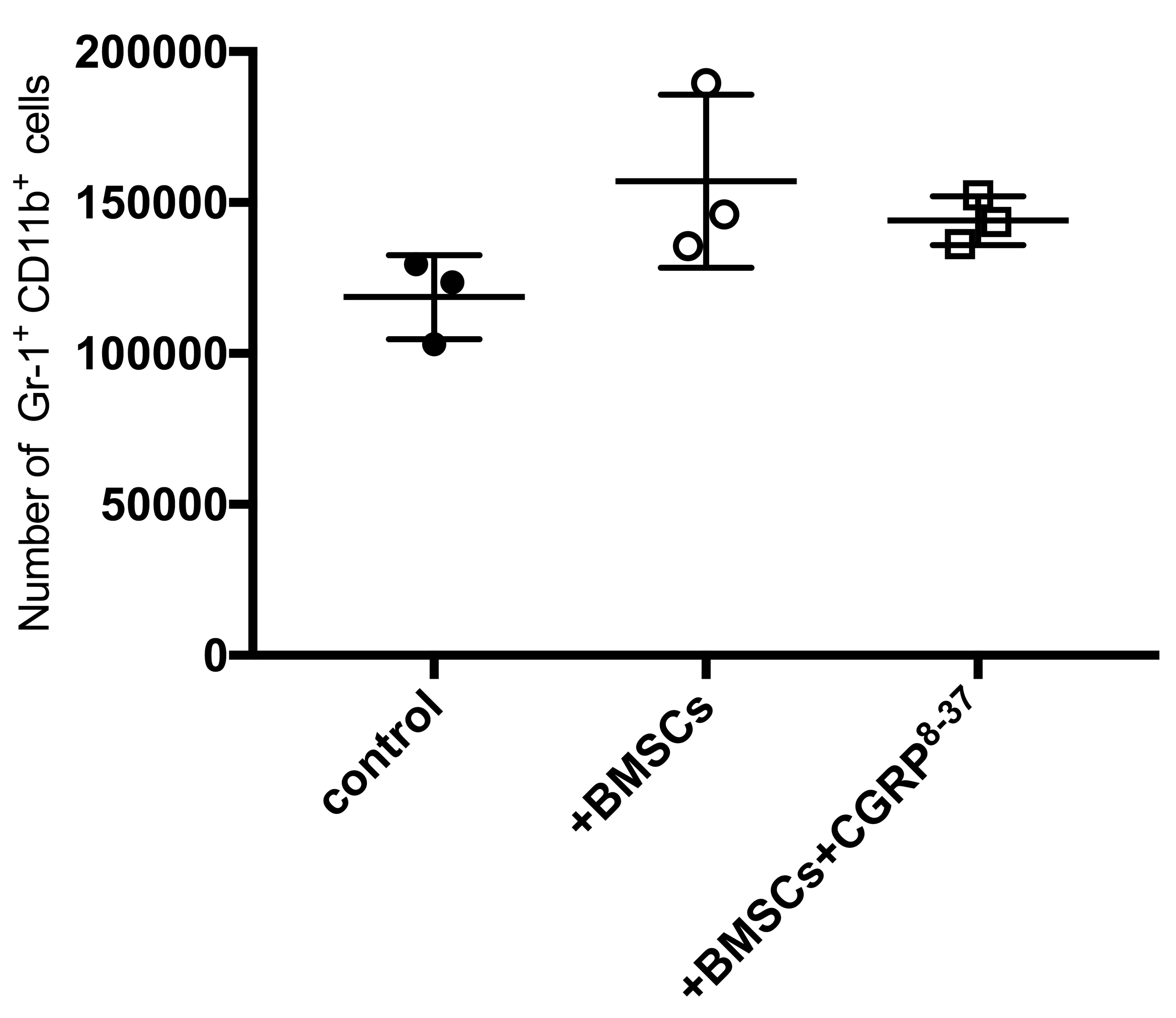


**Differentiated myeloid cell fraction**

**Supplemental Figure S3.** **CGRP signaling from BM stromal cells enhances proliferation of LSK cells.**

The percentages and absolute numbers of Gr-1^+^CD11b^+^ myeloid cells from BMMNCs cultured alone or co-cultured with BM stromal cells in the presence or absence of CGRP^8-37^, as determined by flow cytometery. Data are shown as means ± SD of three mice. **p* < 0.05, ***p* < 0.01 compared with WT mice (Ordinary one-way ANOVA and Turkey’s multiple comparisons test).

**Supplemental Figure S4. Original gels/blots**

**Fig.2a**


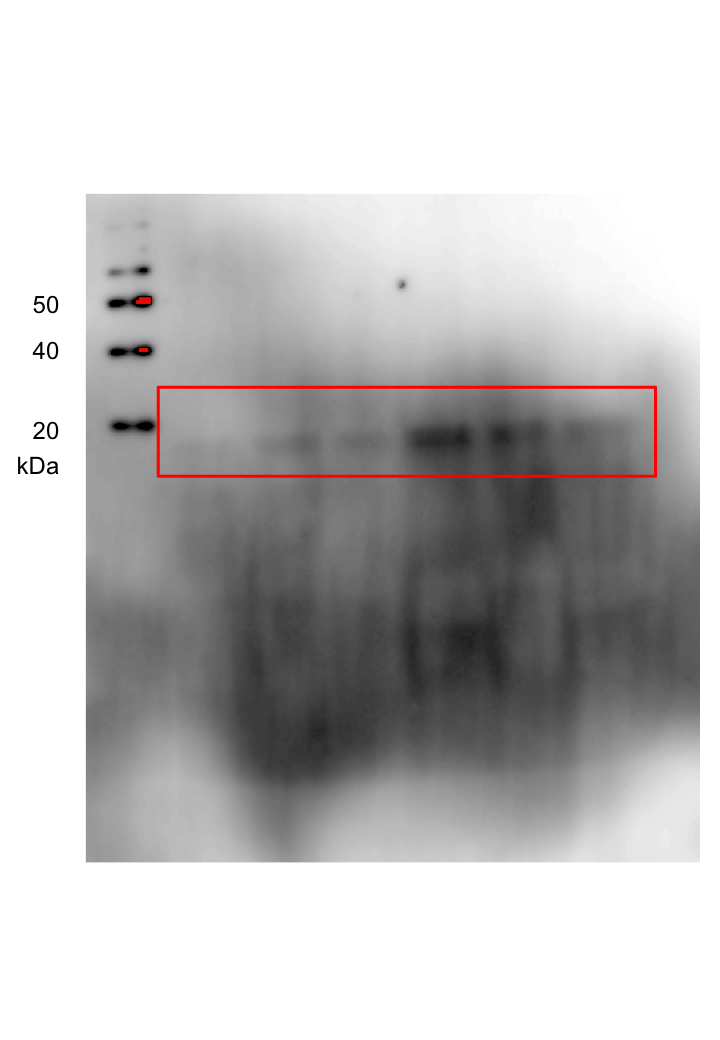


CGRP

**Fig.2a**


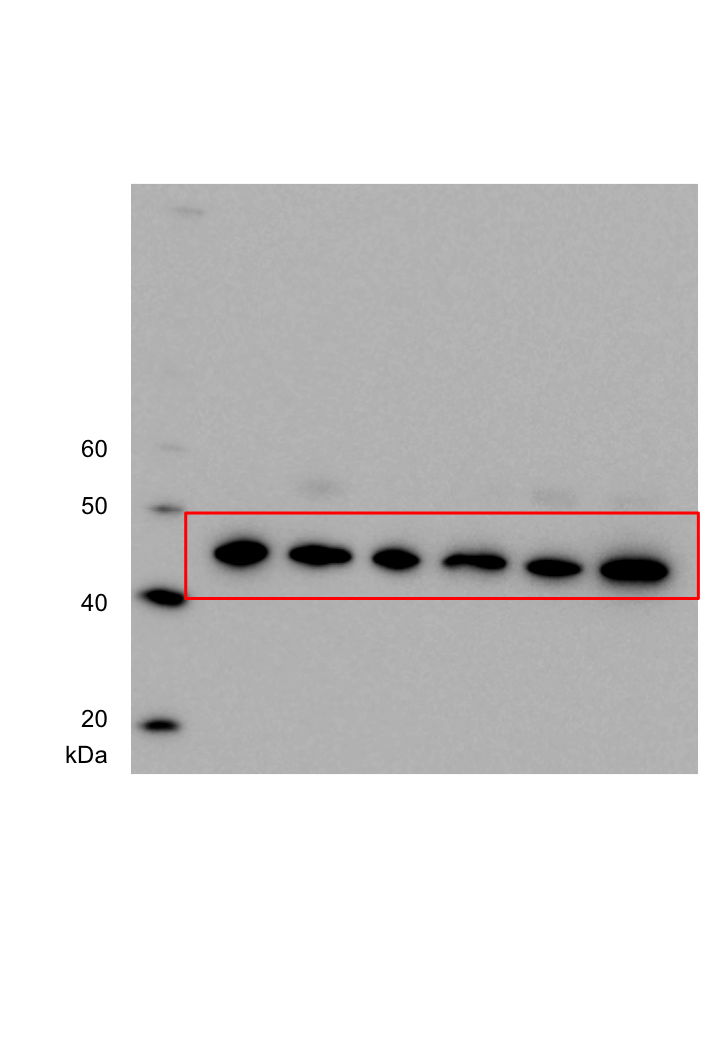


β-actin


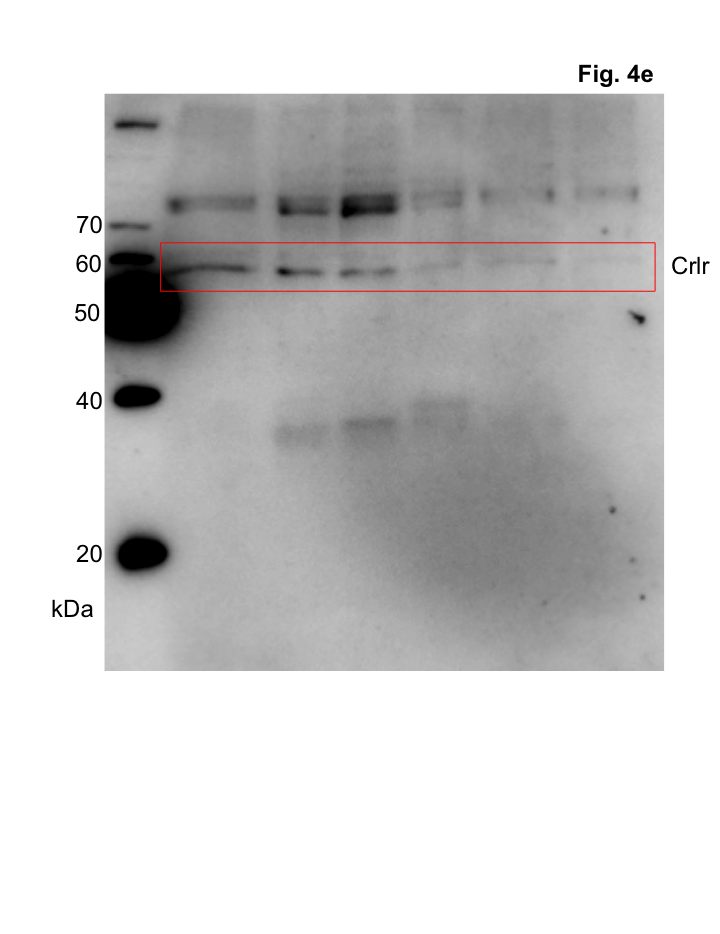


**Fig.4e**


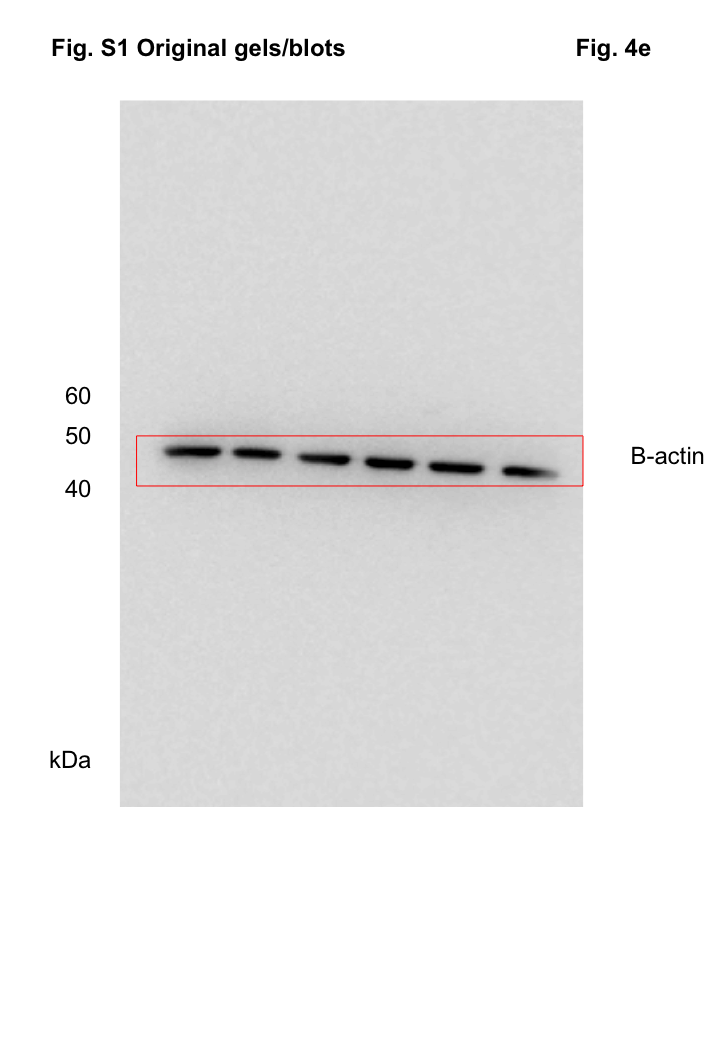


β-actin

**Supplemental Reference**

1. Toda, M. *et al.* Neuronal system-dependent facilitation of tumor angiogenesis and tumor growth by calcitonin gene-related peptide. *Proc. Natl. Acad. Sci. U.S.A.* **105,** 13550–13555 (2008).
